# Antibiotic misuse in respiratory tract infections in children and adults—a prospective, multicentre study (TAILORED Treatment)

**DOI:** 10.1007/s10096-018-03454-2

**Published:** 2019-02-01

**Authors:** Chantal B. van Houten, Asi Cohen, Dan Engelhard, John P. Hays, Roger Karlsson, Edward Moore, David Fernández, Racheli Kreisberg, Laurence V. Collins, Wouter de Waal, Karin M. de Winter-de Groot, Tom F. W. Wolfs, Pieter Meijers, Bart Luijk, Jan Jelrik Oosterheert, Rik Heijligenberg, Sanjay U. C. Sankatsing, Aik W. J. Bossink, Andrew Stubbs, Michal Stein, Sharon Reisfeld, Adi Klein, Ronit Rachmilevitch, Jalal Ashkar, Itzhak Braverman, Valery Kartun, Irena Chistyakov, Ellen Bamberger, Isaac Srugo, Majed Odeh, Elad Schiff, Yaniv Dotan, Olga Boico, Roy Navon, Tom Friedman, Liat Etshtein, Meital Paz, Tanya M. Gottlieb, Ester Pri-Or, Gali Kronenfeld, Einav Simon, Kfir Oved, Eran Eden, Louis J. Bont

**Affiliations:** 1 Division of Paediatric Immunology and Infectious Diseases, University Medical Centre Utrecht, Utrecht University, P.O. Box 85090, Office KC.03.063.0, 3508 AB Utrecht, The Netherlands; 2 MeMed, Tirat Carmel, Israel; 3 Division of Paediatric Infectious Disease Unit, Hadassah-Hebrew University Medical Centre, Jerusalem, Israel; 4 Department of Medical Microbiology and Infectious Diseases, Erasmus University Medical Centre, Rotterdam, The Netherlands; 5 Department of Infectious Diseases, Institute of Biomedicine, Sahlgrenska Academy, University of Gothenburg, Gothenburg, Sweden; 6 Noray Bioinformatics, Derio, Spain; 7 IBEXperts Ltd, Ra’anana, Israel; 8 Department of Paediatrics, Diakonessenhuis, Utrecht, The Netherlands; 9 Department of Paediatric Respiratory Medicine, University Medical Centre Utrecht, Utrecht University, Utrecht, The Netherlands; 10 Department of Paediatrics, Gelderse Vallei Hospital, Ede, The Netherlands; 11 Department of Respiratory Medicine, University Medical Centre Utrecht, Utrecht University, Utrecht, The Netherlands; 12 Department of Internal Medicine and Infectious Diseases, University Medical Centre Utrecht, Utrecht University, Utrecht, The Netherlands; 13 Department of Internal Medicine, Gelderse Vallei Hospital, Ede, The Netherlands; 14 Department of Internal Medicine, Diakonessenhuis Utrecht, Utrecht, The Netherlands; 15 Department of Respiratory Medicine, Diakonessenhuis Utrecht, Utrecht, The Netherlands; 16 Department of Bioinformatics, Erasmus University Medical Centre, Rotterdam, The Netherlands; 17 Department of Paediatrics, Hillel Yaffe Medical Centre, Hadera, Israel; 18 Department of Paediatrics, Bnai Zion Medical Centre, Haifa, Israel; 19 Department of Internal Medicine, Bnai Zion Medical Centre, Haifa, Israel

**Keywords:** Antibiotic use, Pulmonology, Infectious diseases, Respiratory tract infections

## Abstract

Respiratory tract infections (RTI) are more commonly caused by viral pathogens in children than in adults. Surprisingly, little is known about antibiotic use in children as compared to adults with RTI. This prospective study aimed to determine antibiotic misuse in children and adults with RTI, using an expert panel reference standard, in order to prioritise the target age population for antibiotic stewardship interventions. We recruited children and adults who presented at the emergency department or were hospitalised with clinical presentation of RTI in The Netherlands and Israel. A panel of three experienced physicians adjudicated a reference standard diagnosis (i.e. bacterial or viral infection) for all the patients using all available clinical and laboratory information, including a 28-day follow-up assessment. The cohort included 284 children and 232 adults with RTI (median age, 1.3 years and 64.5 years, respectively). The proportion of viral infections was larger in children than in adults (209(74%) versus 89(38%), *p* < 0.001). In case of viral RTI, antibiotics were prescribed (i.e. overuse) less frequently in children than in adults (77/209 (37%) versus 74/89 (83%), *p* < 0.001). One (1%) child and three (2%) adults with bacterial infection were not treated with antibiotics (i.e. underuse); all were mild cases. This international, prospective study confirms major antibiotic overuse in patients with RTI. Viral infection is more common in children, but antibiotic overuse is more frequent in adults with viral RTI. Together, these findings support the need for effective interventions to decrease antibiotic overuse in RTI patients of all ages.

**Electronic supplementary material:**

The online version of this article (10.1007/s10096-018-03454-2) contains supplementary material, which is available to authorized users.

## Introduction

Acute respiratory tract infections (RTIs) are one of the leading causes of emergency department (ED) visits and are often due to viral pathogens [1–4]. Although viral infections are more common in children, studies based on national datasets show that the problem of antibiotic overuse in RTI is largest in adults [4–6]. Unfortunately, it is often not possible to differentiate between viral and bacterial diseases on clinical judgment alone [7]. Antibiotic overuse is associated with an increasing prevalence of antibiotic resistance [8]. In Europe, 25,000 patients die annually due to infections with antibiotic-resistant microorganisms, with estimated costs of €1.5 billion [9–11]. Therefore, there are increasing efforts to study host-biomarkers that could discriminate bacterial from non-bacterial infections [12]. A prospective, international study (The “TAILORED Treatment” (TTT) study) was designed to generate a multi-parametric model for distinguishing between bacterial and viral infections based on new host- or pathogen-related biomarkers [13]. As a gold standard to diagnose bacterial infections is missing, this study used an expert panel reference standard to diagnose each individual patient. Most studies that evaluated antibiotic misuse rates are based on national datasets and classify infections, using general codes, such as the International Classification of Diseases [4–6]. Using guidelines for assessing antibiotic misuse can result in contradictory analyses. For example, Donnelly et al. [4] have classified pharyngitis and tonsillitis as diseases for which antibiotic treatment is appropriate, whereas Barlam et al. [5] have proposed that antibiotic use for these illnesses is inappropriate. Using an expert panel as reference standard has the advantage of individual outcomes (i.e. bacterial or viral infection) for every patients, resulting in more accurate percentages of antibiotic misuse. The current prospective study is aimed to determine antibiotic misuse in children and adults with RTI, using an expert panel reference standard. This study will be instrumental to analyse strategies for new diagnostics to differentiate between viral and bacterial infections.

## Material and methods

### Study design

Patient recruitment for this prospective biomarker TTT-study took place in convenience and consecutive series at the ED and wards of secondary and tertiary hospitals in The Netherlands and Israel [13]. For this subgroup analyses, paediatric patients (aged ≥ 1 month) and adult patients (aged > 18 years), with a suspected upper and/or lower RTI and a maximal disease duration of 8 days, were selected. RTI was defined as presence of two or more of the following signs: tachypnea, cough, nasal flaring, chest retractions, rales, expiratory wheeze and/or decreased breath sounds. For children, WHO age-specific criteria for tachypnea were used [14]. Patients were excluded in case of: previous episode of fever in the past 3 weeks; nosocomial RTI (> 3 days after hospitalisation); psychomotor retardation; moderate-to-severe metabolic disorder; primary or secondary immunodeficiency; proven or suspected HIV, HBV, or HCV infection; and active malignancies. Patients who received antibiotics at any time before the beginning of the study were not excluded. To participate in the study (parental), informed consent was required. The TTT-study is registered on ClinicalTrials.gov, NCT02025699, and was approved by the ethics committees in the participating countries.

### Data collection

Data collection of this TTT-study was described previously [13]. In short, all available clinical data (including biomarkers tested for routine care, a study specific nasal swab and information from a 28-day follow-up assessment) was recorded in an electronic Case Report Form (eCRF) [13]. A multiplex PCR-based assay of the 14 most common respiratory pathogens (nine viruses, five bacteria) was performed on all nasal swabs (MagnaPure LC total nucleic acid kit and MagnaPure 96 DNA, Roche Diagnostics, Mannheim, Germany) [15]. The PCR results were not available for the attending physician, since this assay was performed after completion of the recruitment process.

### Outcomes

Currently, no single reference standard test exists for determining the aetiology of an infection [16]. Therefore, we followed the UK’s National Health Service standard for evaluating diagnostic tests and employed an expert panel reference standard [17]. As described previously, we established expert panels with experienced paediatricians for the paediatric cohort and specialists in internal medicine, pulmonology and infectious diseases for the adult cohort [13]. Every recruited patient was diagnosed by three panel members, and each expert assigned one of the following classifications to each patient: viral infection; bacterial infection; mixed infection (i.e. viral and bacterial co-infection); non-infectious disease; or indeterminate. A majority consensus was applied for the final diagnosis. Patients assigned as ‘mixed infection’ were subsequently classified as bacterial because they are clinically managed similarly. Cases were labelled as ‘inconclusive’ if each panel member assigned a different aetiology or when at least two panel members diagnosed the case as ‘indeterminate’. A microbiologically confirmed diagnosis was predefined as a unanimous panel diagnosis plus the detection of at least one virus for viral cases or for bacterial cases a positive blood culture, excluding the following probable contaminants: coagulase-negative staphylococci; *Corynebacterium* spp.; *Bacillus* spp.; *Propionibacterium acnes*; *Micrococcus* spp*.*; and Viridans group streptococci. For the detection of viruses and bacteria microbiological diagnostics performed for routine care (e.g. blood cultures, sputum cultures and serology) and study, specific nasal swab PCR results were reviewed.

### Statistical analysis

Patients from this convenience cohort of the TTT biomarker study were first stratified according to the reference diagnosis (e.g. viral, bacterial, non-infectious and inconclusive). For the purpose of this study, we excluded non-infectious and inconclusive cases. For the primary objective of this study, we then calculated and compared the percentage of antibiotic use per reference diagnosis for children and adults separately. A sensitivity analysis was performed on the microbiologically confirmed sub-cohort. Secondary analyses were performed for children and adults separately to compare patient characteristics between viral and bacterial infections, antibiotic use per virus, patient characteristics of viral cases receiving and not-receiving antibiotics and different antibiotic agents per country. Sub-cohort analyses were performed for the Dutch and Israeli cohorts separately and for patients admitted to the intensive care unit (ICU). A post hoc analysis was performed on the timing of antibiotic administration in patients with bacterial outcomes to see whether there is delayed antibiotic prescribing (i.e. antibiotics started > 72 h after admission). For baseline characteristics, univariate comparisons were performed using the Fisher exact test, the Student *t* test, and Mann-Whitney test, as appropriate. Statistical analysis was performed using the SPSS version 22.0 for Windows software. A *p* value < 0.05 was considered statistically significant.

## Results

### Patient characteristics

**Fig. 1:**
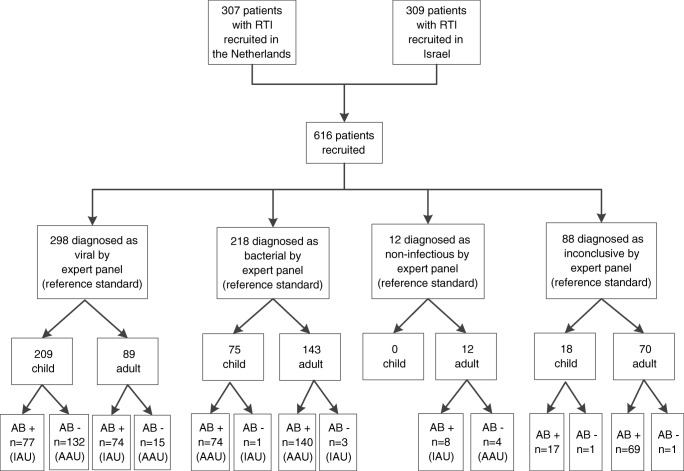
Flowchart of patients AB− antibiotics not prescribed, AB+ antibiotics prescribed, RTI respiratory tract infection, AAU appropriate antibiotic use, IAU inappropriate antibiotic use

Between April 2014 and September 2016, a total of 616 patients with RTI (302 children and 314 adults) were recruited (Fig. 1). The panel diagnosed 516 patients as having a bacterial or a viral infection, encompassing 284 children and 232 adults (median ages, 1.3 years and 64.5 years, respectively) (Table 1). The expert panel diagnosed 12 adults as having a non-infectious disease (predominantly, chronic obstructive pulmonary disease or asthma exacerbation). The reference standard diagnosis was inconclusive for 18 (4 Dutch, 14 Israeli) children and 70 (26 Dutch, 44 Israeli) adults. In 44% of the children with bacterial and viral RTI had comorbidity and not 'bacterial and viral RTI comorbiditis, most of them had mild diseases (e.g. allergies, hyper-reactive airway and eczema). In adults, comorbidity was seen more often (86%) and chronic diseases were more diverse (e.g. cardiovascular risk factors, neurological complaints, pulmonary or cardiac problems). In 215 (76%) children and 120 (52%) adults, the study nasal swab (to help the expert panel establishing the outcome) was positive for one or more microorganisms (Supplemental Table 1). In most of the patients with a bacterial reference standard, a bacterial pathogen was not found (Supplemental Table 1). The study nasal swab was performed in all patients. Therefore, routine care identified significantly fewer pathogens compared to the study swab.

**Table 1:** Baseline of bacterial and viral respiratory tract infections in children and adults. Data are presented as *N* (%), mean (SD), or median [IQR]. LRTI included pneumonia, acute bronchitis and bronchiolitis; URTI included laryngitis, pharyngitis, otitis media, sinusitis, epiglottitis and tonsillitis. Ill-appearing based on attending physician’s impression. *CRP* C-reactive protein, *ICU* intensive care unit, *COPD* chronic obstructive pulmonary disease, *LRTI* lower respiratory tract infection, *URTI* upper respiratory tract infection

	Children (*N* = 284)	Adults (*N* = 232)
Age (years)	1.3 [0.6–3.0]	64.5 [52–75]
Male (sex)	167 (59)	131 (57)
Presence of comorbidity	125 (44)	199 (86)
Ill-appearing	113 (40)	114 (53)
Maximum temperature (°C)	39.2 (0.9)	38.6 (1.0)
Duration of symptoms (days)	3 (2)	4 (2)
Hospital admission	208 (75)	217 (94)
Hospitalisation duration, days	4 [3–8]	5 [3–8]
CRP (mg/L) at admission	16 [4–43]	34 [9–136]
Disease severity		
Oxygen saturation (%)	95 [92–98]	94 [91–96]
Needed mechanical ventilation	31 (11)	3 (1)
Deaths	1 (1)	3 (1)
Admission site		
Secondary care centre	198 (70)	173 (75)
Tertiary care centre	47 (16)	53 (23)
ICU	39 (14)	6 (2)
Country		
The Netherlands	136 (48)	131 (56)
Israel	148 (52)	101 (44)
Clinical syndrome		
COPD/asthma exacerbation	4 (1)	45 (19)
LRTI	150 (53)	172 (74)
URTI	130 (46)	15 (7)

### Patient outcomes

The proportion of viral infections was larger in children than in adults (209/284 (74%) versus 89/232 (38%), respectively, *p* < 0.001). Most bacterial co-infections were observed in children infected with rhinovirus (17/62, 27%) and respiratory syncytial virus (RSV) (23/98, 23%), whereas influenza was most frequently associated with bacterial co-infection in adults (17/52, 33%, Table 2). Children and adults with bacterial infections were more often hospitalised (*p* values respectively < 0.0001 and 0.009) and had higher CRP values (*p* value 0.001 and < 0.0001 respectively) compared with patients with a viral infection (Table 3). In 172/284 (61%) of the paediatric cohort and 114/232 (49%) of the adult patients, the expert panel diagnosis can be confirmed microbiologically. This microbiologically confirmed sub-cohort includes in total 286 patients, 145 children and 58 adults with viral infection and 27 children and 56 adults with bacterial infection (Supplemental Fig. 1).

**Table 2:** Appropriate and inappropriate antibiotic usage per virus. a. Paediatric cohort. b. Adult cohort. Viral and bacterial diagnoses based on expert panel diagnoses. Mixed infection was considered as bacterial. Data shown represent the numbers of positive PCR of nasal swabs performed for the study and *N* (%) of patients in this group receiving antibiotics. *RSV* respiratory syncytial virus

a.				
Paediatric	Viral *N* = 209		Bacterial *N* = 75	
	Viruses detected^a^	Antibiotic use^c^	Viruses detected	Antibiotic use
Adenovirus	28	12(43)	2	2(100)
Bocavirus	22	7(32)	5	5(100)
Influenza virus	30	10(33)	6	6(100)
Rhinovirus	45	16(36)	17	16(94)
RSV	75	32(43)	23	22(96)
Other^b^	26	11(42)	8	8(100)
b.				
Adult	Viral *N* = 89		Bacterial *N* = 143	
	Viruses detected	Antibiotic use^c^	Viruses detected	Antibiotic use
Influenza virus	35	30(86)	17	16(94)
Rhinovirus	16	12(75)	6	6(100)
RSV	14	13(93)	4	4(100)
Other^d^	11	10(91)	8	8(100)

^a^As some patients tested positive for more than one virus, the total number of detected viruses is higher than the number of patients. ^b^Includes coronavirus, human metapneumovirus, and parainfluenza virus. ^c^Numbers of antibiotic usages are given per virus. As some patients tested positive for more than one virus, the total antibiotic usage is different with respect to the numbers given in Fig. 1. ^d^Includes adenovirus, bocavirus, coronavirus, human metapneumovirus and parainfluenza virus

**Table 3:** Comparison of patients with viral and bacterial reference standards. a. Paediatric cohort. b. Adult cohort. Viral and bacterial diagnoses based on expert panel diagnoses. Mixed infection was considered as bacterial. Data are presented as *N* (%), mean (SD), or median [IQR]. *CRP* C-reactive protein, *ICU* intensive care unit, *COPD* chronic obstructive pulmonary disease, *LRTI* lower respiratory tract infection, *URTI* upper respiratory tract infection

a. Paediatric cohort	Viral *N* = 209	Bacterial *N* = 75	*p* value
Age (years)	1.2 [0.6–2.8]	1.3 [0.5–5.8]	0.102
Male sex	119 (57)	48 (64)	0.122
Presence of comorbidity	86 (41)	39 (52)	0.104
Ill-appearing	75 (36)	38 (51)	0.059
Maximum temperature (°C)	39.1 (0.9)	39.3 (0.9)	0.150
Duration of symptoms (days)	3 (2)	3 (2)	0.497
Hospital admission	144 (70)	64 (91)	< 0.0001
Hospitalisation duration (days)	4 [3–6]	4 [2–16]	0.050
CRP (mg/L) at admission	13 [4–38]	22 [6–131]	0.001
Oxygen saturation (%)	96 [92–98]	95 [91–98]	0.523
Need mechanical ventilation	12 (6)	19 (25)	< 0.0001
Admission site			< 0.0001
Secondary care centre	152 (73)	46 (61)	
Tertiary care centre	40 (19)	7 (9)	
ICU	17 (8)	22 (29)	
Country			0.291
The Netherlands	104 (50)	32 (43)	
Israel	105 (50)	43(57)	
Clinical syndrome			< 0.0001
Asthma exacerbation	4 (2)	0 (0)	
LRTI	110 (53)	55 (73)	
URTI	95 (45)	20 (27)	
b. Adult cohort	Viral *N* = 89	Bacterial *N* = 143	*p* value
Age (years)	61 [46–72]	67 [53–75]	0.061
Male sex	46 (52)	85 (59)	0.247
Presence of comorbidity	79 (89)	120 (84)	0.304
Ill-appearing	38 (43)	76 (59)	0.023
Maximum temperature (°C)	38.3 (0.9)	38.7 (1.0)	0.015
Duration of symptoms (days)	4 (2)	4 (3)	0.495
Hospital admission	79 (89)	138 (97)	0.009
Hospitalisation duration (days)	4 [3–6]	6 [3–9]	0.010
CRP (mg/L) at admission	14 [4–43]	67 [16–193]	< 0.0001
Oxygen saturation (%)	95 [91–96]	94 [92–97]	0.779
Needed mechanical ventilation	2 (2)	1 (1)	0.310
Admission site			0.007
Secondary care centre	71 (80)	102 (71)	
Tertiary care centre	13 (15)	40 (28)	
ICU	5 (5)	1 (1)	
Country			0.376
The Netherlands	47 (53)	84 (59)	
Israel	42 (47)	59 (41)	
Clinical syndrome			0.001
COPD/asthma exacerbation	23 (26)	22 (15)	
LRTI	55 (62)	117 (82)	
URTI	11 (12)	4 (3)	

### Antibiotic usage

The overall antibiotic prescription rate for viral and bacterial RTI was 71%, and the antibiotic overuse rate (i.e. antibiotic prescription for viral RTI) was 51%. Antibiotics were administered less frequently to children than adults with a viral infection (77/209 (37%) versus 74/89 (83%), *p* < 0.001, (Fig. 1). This difference was similar across different viral pathogens, including influenza and RSV (Table 2). Within the microbiologically confirmed sub-cohort, similar percentages of antibiotic overuse were observed (50/145 (34%) children versus 50/58 (86%) adults (Supplemental Fig. 1). Children receiving antibiotics for viral RTI were more often admitted to the ICU (*p* value 0.032) and had more often lower RTI (*p* value 0.001), compared with children not receiving antibiotics (Table 4). Adults with viral RTI receiving antibiotics were more often male (*p* value 0.033), had higher temperatures (*p* value 0.004) and also had more often lower RTI (*p* value 0.003), compared with adults not receiving antibiotics (Table 4). Among the patients with bacterial RTI (*n* = 218), only one (1%) child and three (2%) adults were not treated with antibiotics (Supplemental Table 2). Dutch children received mostly amoxicillin/clavulanate, whereas Israeli children received mostly amoxicillin. Among adults, the most prescribed antibiotic agents were amoxicillin (The Netherlands) and roxithromycin (Israel, Supplemental Fig. 2). From patients with bacterial outcome, information on antibiotic timing was available for 107 patients (49%). In eight children (7%), antibiotics were prescribed > 72 h after admission; seven of these children were admitted on the ICU. All adults received antibiotics within 72 h after presentation.

**Table 4:** Baseline of viral respiratory tract infections children and adults, antibiotics versus no antibiotics. a. Paediatric cohort. b. Adult cohort. Data are presented as *N* (%), mean (SD), or median [IQR]. LRTI included pneumonia, acute bronchitis and bronchiolitis; URTI included laryngitis, pharyngitis, otitis media, sinusitis and tonsillitis. *AB+* antibiotics prescribed, *AB*− antibiotics not prescribed, *CRP* C-reactive protein, *ICU* intensive care unit, *COPD* chronic obstructive pulmonary disease, *LRTI* lower respiratory tract infection, *URTI* upper respiratory tract infection

a.	AB+ (*N* = 77)	AB− (*N* = 132)	*p* value
Age (years)	1.0 [0.5–2.7]	1.2 [0.6–2.8]	0.945
Male sex	42 (55)	77 (58)	0.594
Presence of comorbidity	24 (31)	62 (47)	0.025
Ill-appearing	30 (39)	45 (35)	0.473
Maximum temperature (°C)	39.2 (0.9)	39.1 (0.8)	0.479
Duration of symptoms (days)	3 (2)	3 (2)	0.352
Hospital admission	60(79)	84 (65)	0.030
Hospitalisation duration (days)	5 [3–9]	3 [2–4]	< 0.001
CRP (mg/L) at admission	14 [3–32]	10 [3–26]	0.294
Disease severity			
Oxygen saturation, %	95 [88–97]	97 [93–99]	0.051
Needed mechanical ventilation	9 (12)	3 (2)	0.005
Death	0 (0)	0 (0)	NA
Admission site			0.032
Secondary care centre	50 (65)	102 (77)	
Tertiary care centre	16 (21)	24 (18)	
ICU	11 (14)	6 (5)	
Country			0.070
The Netherlands	32 (42)	72 (55)	
Israel	45 (58)	60 (45)	
Clinical syndrome			0.001
COPD/asthma exacerbation	0 (0)	4 (3)	
LRTI	48 (62)	47 (36)	
URTI	29 (38)	81 (61)	
b.	AB+ (*n* = 74)	AB− (*n* = 15)	*p* value
Age (years)	64 [47–75]	56 [51–60]	0.086
Male sex	42 (57)	4 (27)	0.033
Presence of comorbidity	66 (89)	13 (87)	0.778
Ill-appearing	34 (47)	4 (27)	0.156
Maximum temperature (°C)	38.5 (0.9)	37.8 (0.6)	0.004
Duration of symptoms (days)	4 (2)	3 (2)	0.478
Hospital admission	66 (89)	13 (87)	0.778
Hospitalisation duration (days)	4 [3–6]	4 [2–7]	0.805
CRP (mg/L) at admission	15 [5–45]	7 [3–35]	0.332
Disease severity			
Oxygen saturation (%)	95 [91–96]	95 [91–98]	0.317
Needed mechanical ventilation	2 (3)	0 (0)	0.520
Death	1 (1)	0 (0)	0.651
Admission site			0.234
Secondary care centre	60 (81)	11 (73)	
Tertiary care centre	9 (12)	4 (27)	
ICU	5 (7)	0 (0)	
Country			0.021
The Netherlands	35 (47)	12 (80)	
Israel	39 (53)	3 (20)	
Clinical syndrome			0.003
COPD/asthma exacerbation	14 (19)	9 (60)	
LRTI	51 (69)	4 (27)	
URTI	9 (12)	2 (13)	

### Subgroup analysis

We analysed the Dutch (*n* = 267) and Israeli (*n* = 249) cohorts separately (Supplemental Table 3). The children and adults in the Dutch cohort more often had comorbidity, had higher CRP concentrations and more often needed mechanical ventilation compared to the Israeli patients. The proportion of bacterial infections was similar in both countries. Antibiotic overuse in children with viral infections was similar in the Dutch and Israeli cohorts (32/104 (31%) versus 45/105 (43%), *p* = 0.07). In adults with viral infection, the proportion of patients receiving antibiotics was lower in The Netherlands, when compared with Israel (35/47 (74%) versus 39/42 (93%), *p* = 0.021). Of all 284 children, 39 (14%) children were admitted to the ICU. Thirty-three (85%) ICU patients received antibiotics, 11 (33%) had viral infection. Six (2%) adults were admitted to the ICU; all received antibiotics. Five adult ICU patients had viral infections, and one patient had a bacterial infection. Influenza virus was detected in four of them.

## Discussion

This convenience cohort of patients from the TTT biomarker study is the first prospective study comparing the burden of antibiotic misuse in both children and adults diagnosed with RTI, using an expert panel adjudication as the reference standard [18]. We observed that antibiotic overuse was less common in children than in adults with a viral RTI (37% versus 83%), regardless of viral aetiology. Only one (1%) child and three (2%) adults with bacterial infection were not treated with antibiotics (i.e. underuse); all “untreated patients” were mild cases with full spontaneous recovery.

As mentioned before, most studies that evaluated antibiotic misuse rates are based on national datasets and classify infections, using general codes, such as the International Classification of Diseases [4–6]. In the present prospective study, using an expert panel reference standard, we confirmed that antibiotic overuse in viral RTI is more prevalent among adult patients. In our study, children less often had comorbidity, appeared to be less ill at presentation and had lower a priori probabilities of having a bacterial infection, compared with adults. Physicians are more inclined to initiate antibiotic treatment if the patient appears to be ill upon presentation, even if a viral pathogen was detected, and do often not adhere to related recommendations [19, 20]. Therefore, in addition to effective diagnostic tests, education and prescribing feedback are needed to reduce antibiotic overuse [21, 22].

The percentages of antibiotic underuse in our study were low. In literature, underuse up to 31% for children with pneumonia has been described [23, 24]. Therefore, we performed a post hoc analysis on a selection of patients for whom information about the timing of antibiotic administration was available. We found delayed antibiotic prescribing (i.e. antibiotics started > 72 h after admission) in only seven children who were admitted to the ICU and one non-ICU child; there were no delayed antibiotic prescriptions in adults. The expert panel may have underestimated bacterial infections in patients recovering without antibiotics.

We included data from two different countries, both with different healthcare systems, to make the results of this study more generalizable. We did not observe significant differences in overall antibiotic use between Dutch and Israeli children and adults. However, existing literature shows that antibiotic use is higher in Israel, compared with The Netherlands [25]. A relatively high rate of antibiotic use in The Netherlands may be related to the high proportion of severely ill patients (e.g. more bacterial infection, more ICU admissions) in the Dutch cohort.

A strength of our study is that the cohort comprised both children and adults, enabling a direct comparison of findings without any confounding issues related to the methodology. A second strength is the thorough nature of our reference standard to distinguish viral from bacterial infections [16, 26]. Clinical suspicion confirmed by microbiological results is an approach often employed in other studies as a reference standard, although this method can restrict the analysis to the easy-to-diagnose patients and is not always technically applicable to RTIs. Using an expert panel has the advantage of capturing a wider spectrum of illness severities and, therefore, is more likely to be generalizable to clinical practice [16, 27]. The expert panel was provided with all available clinical information, including information about the course of the disease, all microbiological results (including study-specific multiplex PCR on nasal swabs), and information from a 28-day follow-up assessment. These information were not available to the attending physicians when deciding to start antibiotics or not.

A limitation of this study is that not all eligible patients participated in this study for practical reasons (e.g. attending physician did not have time to recruit the patient at the ED, parents or patients did not want phlebotomy only for study proposes), which may have introduced a selection bias in favour of more severely ill patients and could lead to an overestimation of antibiotic overuse. A second limitation is that, by design, patients with an inconclusive panel diagnosis (*n* = 88) were excluded, although it is notable that 98% of whom were receiving antibiotics. Therefore, including these patients would probably not change the results. Third, we collected a nasal swab for every patient for the establishment of patient diagnosis; other microbiological diagnostics (e.g. sputum and blood cultures) were only performed if indicated for routine care. Standardising more microbiological diagnostics might have led to fewer inconclusive panel diagnoses. A fourth limitation is that we do not have information on the use of influenza and pneumococcal vaccines available. As a consequence, we cannot exclude that information on vaccine history of participants would have allowed for a more accurate panel diagnosis. Fifth, we cannot exclude that some possible confounders (e.g. comorbidity, hospital admission and site-specific protocols) might drive some of the difference in prescribing practices between children and adults. Sixth, this study is a sub-analysis of a convenience cohort of the TTT biomarker study. Therefore, no sample size calculation for this objective was made. Seventh, the presented proportions of antibiotic misuse are based on expert panel diagnoses using all available information after 28 days. We do not have a reference standard diagnosis at the moment of presentation, and therefore, analyses regarding antibiotic misuse using the current available diagnostic tests could not be performed. Eight, the eCRF used in this study does not include information regarding negative microbiological test results. Ninth, the inclusion criteria used in this study mostly includes symptoms of lower RTI. This probably leads to an underestimation of the proportion of patients with an upper RTI. However, several patients did have symptoms of an upper RTI, and therefore, we believe that the study cohort is representative for daily practice. Finally, defined daily antibiotic dosages per 1000 patient days in France, Greece, the UK and the USA is 1.5–3.3 times higher than in The Netherlands [28]. Due to the low antibiotic prescription rates in The Netherlands, it is plausible that antibiotic overuse will be even higher in other countries.

In conclusion, viral RTI is more common in children, whereas antibiotic overuse is more common in adult patients with RTI, supporting the need for better diagnostics to differentiate between viral and bacterial infection across all ages.

## Electronic supplementary material

ESM 1 (DOCX 124 kb)

## Data Availability

The datasets generated during and/or analysed during the current study are available from the corresponding author on reasonable request.
