# Supplementary material for: Antibiotic misuse in respiratory tract infections in children and adults—a prospective, multicentre study (TAILORED Treatment)
Source: Eur J Clin Microbiol Infect Dis. 2019 Feb 1;38(3):505–14. doi: 10.1007/s10096-018-03454-2 (PMC6394715; doi:10.1007/s10096-018-03454-2)
Supplement: Supplementary file 1 — (DOCX 124 kb) [file 10096_2018_3454_MOESM1_ESM.docx]

**Supplementary material**

Antibiotic misuse in respiratory tract infections in children and adults – a prospective, multicentre study (TAILORED Treatment)

European Journal of Clinical Microbiology & Infectious Diseases.

Chantal B. van Houten, Asi Cohen, Dan Engelhard, John P. Hays, Roger Karlsson, Edward Moore, David Fernández, Racheli Kreisberg, Laurence V. Collins, Wouter de Waal, Karin M. de Winter-de Groot, Tom F.W. Wolfs, Pieter Meijers, Bart Luijk, Jan Jelrik Oosterheert, Rik Heijligenberg, Sanjay U.C. Sankatsing, Aik W.J. Bossink, Andrew Stubbs, Michal Stein, Sharon Reisfeld, Adi Klein, Ronit Rachmilevitch, Jalal Ashkar, Itzhak Braverman, Valery Kartun, Irena Chistyakov, Ellen Bamberger, Isaac Srugo, Majed Odeh, Elad Schiff, Yaniv Dotan, Olga Boico, Roy Navon, Tom Friedman, Liat Etshtein, Meital Paz, Tanya M. Gottlieb, Ester Pri-Or, Gali Kronenfeld, Einav Simon, Kfir Oved, Eran Eden, Louis J. Bont

**Corresponding author**: Louis Bont, MD, PhD, University Medical Centre Utrecht, Paediatric Immunology and Infectious Diseases, P.O. Box 85090, Office KC.03.063.0, 3508 AB Utrecht, the Netherlands, Tel.: +31 88 75 540 03, Fax: +31 88 75 553 50, [l.bont@umcutrecht.nl](mailto:l.bont@umcutrecht.nl)

**Supplemental Table 1 Microbiology results**

a. Paediatric cohort. b. Adult cohort. Microbiology results from study specific nasal swab PCR (performed on all patients) and positive results from microbiology diagnostics performed for routine care. Reference standard outcomes are based on the majority consensus of the expert panel. Mixed infection was considered as ‘bacterial’. Data are presented as N (%).*Some patients tested positive for different candida pathogens, seven adults tested positive for candida.

**a.**

|  | Total  N=284 | Viral  infection  N=209 | Bacterial  infection  N=75 |
| --- | --- | --- | --- |
| Study nasal swab PCR  (performed on all patients) |  |  |  |
| 0 microorganism | 69(24) | 44(21) | 25(33) |
| 1 microorganism | 151(53) | 114(55) | 37(50) |
| 2 microorganisms | 48(17) | 38(18) | 10(13) |
| 3 microorganisms | 16(6) | 13(6) | 3(4) |
| Microorganism |  |  |  |
| Adenovirus | 30(11) | 28(13) | 2(3) |
| Bocavirus | 27(10) | 22(11) | 5(7) |
| Coronavirus | 13(5) | 8(4) | 5(7) |
| Influenza virus A/B | 36(13) | 30(14) | 6(8) |
| Metapneumovirus | 11(4) | 9(4) | 2(3) |
| Parainfluenza virus | 10(4) | 9(4) | 1(1) |
| Respiratory syncytial virus A/B | 98(35) | 75(36) | 23(31) |
| Rhinovirus A/B/C | 62(22) | 45(22) | 17(23) |
| Mycoplasma pneumonia | 3(1) | 1(0.5) | 2(5) |
| *Bordetella pertussis* | 3(1) | 0(0) | 3(7) |
| *Bordetella parapertussis* | 1(0.5) | 0(0) | 1(2) |
|  |  |  |  |
| Routine care |  |  |  |
| 0 microorganism | 220(77) | 174(83) | 46(61) |
| 1 microorganism | 41(15) | 27(13) | 14(19) |
| 2 microorganisms | 14(5) | 8(4) | 6(8) |
| 3 microorganisms | 7(2) | 0(0) | 7(9) |
| 4 microorganisms | 2(1) | 0(0) | 2(3) |
| Positive blood culture |  |  |  |
| Staphylococcus, coagulase-negative | 2(1) | 1(0.5) | 1(1) |
| *Streptococcus pneumoniae* | 1(0.5) | 0(0) | 1(1) |
| *Haemophilus influenzae* | 2(1) | 0(0) | 2(3) |
| *Fusobacterium necrophorum* | 1(0.5) | 0(0) | 1(1) |
| Positive serology |  |  |  |
| Respiratory syncytial virus A/B | 3(1) | 2(1) | 1(1) |
| Positive sputum culture/PCR |  |  |  |
| Respiratory syncytial virus A/B | 12(4) | 6(3) | 6(8) |
| *Moraxella catarrhalis* | 11(4) | 1(0.5) | 10(13) |
| *Haemophilus influenzae* | 9(3) | 2(1) | 7(9) |
| *Streptococcus pneumoniae* | 5(2) | 0(0) | 5(7) |
| *Enterobacter cloacae* | 4(1) | 1(0.5) | 3(4) |
| Group A streptococcus | 1(0.5) | 1(0.5) | 0(0) |
| *Pseudomonas aeruginosa* | 1(0.5) | 0(0) | 1(1) |
| Positive nasopharyngeal culture/PCR |  |  |  |
| Group A streptococcus | 5(2) | 1(0.5) | 4(5) |
| *Streptococcus pneumoniae* | 1(0.5) | 1(0.5) | 0(0) |
| *Haemophilus influenzae* | 1(0.5) | 1(0.5) | 0(0) |
| *Mycoplasma pneumoniae* | 1(0.5) | 0(0) | 1(1) |
| *Bordetella pertussis* | 1(0.5) | 0(0) | 1(1) |
| Respiratory syncytial virus A/B | 19(7) | 13(6) | 6(8) |
| Adenovirus | 5(2) | 3(1) | 2(5) |
| Influenza virus | 5(2) | 4(2) | 1(1) |
| Rhinovirus | 3(1) | 1(0.5) | 2(5) |
| Metapneumovirus | 3(1) | 3(1) | 0(0) |
| Coronavirus | 1(0.5) | 1(0.5) | 0(0) |
| Parainfluenza virus | 1(0.5) | 1(0.5) | 0(0) |

**b.**

|  | Total  N=232 | Viral  infection  N=89 | Bacterial  infection  N=143 |
| --- | --- | --- | --- |
| Study nasal swab PCR  (performed on all patients) |  |  |  |
| 0 microorganism | 112(48) | 17(19) | 95(66) |
| 1 microorganism | 115(50) | 68(76) | 47(33) |
| 2 microorganisms | 5(2) | 4(5) | 1(1) |
| Microorganism |  |  |  |
| Adenovirus | 3(1) | 2(2) | 1(1) |
| Bocavirus | 1(0.5) | 0(0) | 1(1) |
| Coronavirus | 4(2) | 1(1) | 3(2) |
| Influenza virus A/B | 52(22) | 35(39) | 17(12) |
| Metapneumovirus | 6(3) | 4(5) | 2(1) |
| Parainfluenza virus | 5(2) | 4(5) | 1(1) |
| Respiratory syncytial virus A/B | 18(8) | 14(16) | 4(3) |
| Rhinovirus A/B/C | 22(10) | 16(18) | 6(4) |
| *Mycoplasma pneumoniae* | 11(5) | 0(0) | 11(8) |
| *Bordetella pertussis* | 3(1) | 0(0) | 3(2) |
|  |  |  |  |
| Routine care |  |  |  |
| 0 microorganism | 140(60) | 55(62) | 85(60) |
| 1 microorganism | 79(34) | 32(36) | 47(33) |
| 2 microorganisms | 8(4) | 1(1) | 7(5) |
| 3 microorganisms | 3(1) | 1(1) | 2(1) |
| 4 microorganisms | 2(1) | 0(0) | 2(1) |
| Positive blood culture/PCR |  |  |  |
| *Streptococcus pneumoniae* | 5(2) | 0(0) | 5(3) |
| *Moraxella osloensis* | 1(0.5) | 0(0) | 1(1) |
| *Chlamydophila pneumoniae* | 1(0.5) | 0(0) | 1(1) |
| Positive serology |  |  |  |
| *Bordetella pertussis* | 1(0.5) | 0(0) | 1(1) |
| *Mycoplasma pneumoniae* | 1(0.5) | 0(0) | 1(1) |
| CMV | 1(0.5) | 1(1) | 0(0) |
| EBV | 1(0.5) | 1(1) | 0(0) |
| *Mycobacterium tuberculosis* | 1(0.5) | 0(0) | 1(1) |
| Positive sputum culture |  |  |  |
| *Haemophilus influenzae* | 15(6) | 1(1) | 14(10) |
| *Moraxella catarrhalis* | 3(1) | 0(0) | 3(2) |
| *Serratia ureilytica* | 1(0.5) | 0(0) | 1(1) |
| *Staphylococcus aureus* | 5(2) | 1(1) | 4(3) |
| *Streptococcus pneumoniae* | 4(1.5) | 0(0) | 4(3) |
| *Acinetobacter baumannii* | 3(1) | 0(0) | 3(2) |
| *Candida albicans** | 5(2) | 0(0) | 5(3) |
| *Candida tropicalis** | 3(1) | 1(1) | 2(1) |
| *Candida glabrata** | 2(1) | 0(0) | 2(1) |
| *Klebsiella oxytoca* | 1(0.5) | 0(0) | 1(1) |
| *Pseudomonas aeruginosa* | 5(2) | 1(1) | 4(3) |
| Positive nasopharyngeal culture/PCR |  |  |  |
| Group A streptococcus | 1(0.5) | 0(0) | 1(1) |
| *Staphylococcus aureus* | 1(0.5) | 0(0) | 1(1) |
| *Mycoplasma pneumoniae* | 7(3) | 0(0) | 7(5) |
| *Candida albicans* | 2(1) | 1(1) | 1(1) |
| Metapneumovirus | 4(1.5) | 3(3) | 1(1) |
| Respiratory syncytial virus A/B | 4(1.5) | 3(3) | 1(1) |
| Adenovirus | 3(1) | 2(2) | 1(1) |
| Rhinovirus | 3(1) | 3(3) | 0(0) |
| Influenza virus | 26(11) | 18(20) | 8(6) |
| Parainfluenza virus | 1(0.5) | 1(1) | 0(0) |

**Supplemental Table 2 Cases with a reference standard diagnosis of bacterial infection not treated with antibiotics**

CRP: C-reactive protein, F: female, M:male, NL: The Netherlands, IL: Israel, COPD: Chronic Obstructive Pulmonary Disease, DM2: Diabetes Mellitus Type 2, TIA: Transient Ischemic Attack

| **Pt.** | **Clinical**  **syndrome** | **Age (y)** | **Sex** | **Country** | **Maximal temperature**  **(°C)** | **CRP** | **Hospitalization duration (d)** | **Comorbidity** | **Microbiology** | **Clinical details** |
| --- | --- | --- | --- | --- | --- | --- | --- | --- | --- | --- |
| **Children** | | | | | | | | | | |
| 1 | Bronchiolitis | 0.25 | M | IL | 37.4 | 21 | 3 | Pulmonary valve stenosis | Nasal swab: *Bordetella parapertussis*, RSV and rhinovirus. | Presented with cough and dyspnea since 5 days. Clinical examination: bilateral lymphadenopathy cervical, crepitation, wheeze and systolic murmur. Chest X-ray: normal. |
| **Adults** | | | | | | | | | | |
| 1 | COPD Exacerbation | 66 | F | NL | 36.8 | 5 | 9 | DM2, COPD, TIA, schizophrenic, fibromyalgia | Sputum culture: *Haemophilus influenzae* | Presented with cough and dyspnea since 7 days. Clinical examination: wheeze and prolonged expiration. Chest X-ray: normal. Required oxygen even after discharge. |
| 2 | COPD Exacerbation | 40 | M | NL | 38.8 | 6 | 7 | COPD, asthma, dust allergy, addiction to cigarettes, heroin and cocaine | Sputum culture: *Streptococcus pneumonia, Citrobacter koseri, Candida albicans,* Influenza virus. Blood culture: negative | Presented with dyspnea, dry cough, headache and myalgia since 4 days. Clinical examination: wheeze only. Chest X-ray: normal (twice).  Required oxygen for 3 days. |
| 3 | COPD Exacerbation | 47 | M | IL | 36.5 | 1 | 1 | None | Bronchoalveolar lavage: *Staphylococcus aureus* | Presented with dyspnea and cough since 14 days. CT-chest: Infiltrative inflammation and consolidation.  During admission, COPD was diagnosed. |

**Supplemental Table 3 Baseline of bacterial and viral RTI children and adults, the Netherlands versus Israel**

Data are presented as n (%), mean (SD), or median [IQR]. LRTI included pneumonia, acute bronchitis and bronchiolitis; URTI included laryngitis, pharyngitis, otitis media, sinusitis and tonsillitis. NL: The Netherlands, IL: Israel, CRP: C-reactive protein, ICU: intensive care unit, COPD: Chronic Obstructive Pulmonary Disease, LRTI: lower respiratory tract infection, URTI: upper respiratory tract infection, RSV: respiratory syncytial virus.

|  | Children | | Adults | |
| --- | --- | --- | --- | --- |
|  | **NL**  **(N=136)** | **IL**  **(N=148)** | **NL**  **(N=131)** | **IL**  **(N=101)** |
| Age, years | 0.9[0.2-3.1] | 1.7[0.9-3.0] | 66[56-74] | 61[40-76] |
| Male sex | 81(60) | 86(58) | 71(54) | 60(59) |
| Presence of comorbidity | 74(54) | 51(35) | 122(93) | 77(76) |
| Ill-appearing | 77(58) | 36(24) | 50(42) | 64(66) |
| Maximal temperature, °C | 39,1(0,8) | 39,3(0,9) | 38,7(0,9) | 38,4(1,1) |
| Duration of symptoms, days | 3(2) | 3(2) | 3(2) | 4(3) |
| Hospital admission | 125(92) | 83(59) | 124(95) | 93(93) |
| Hospitalization duration, days | 5[3-14] | 3[2-4] | 6[4-9] | 3[2-6] |
| CRP (mg/L) at admission | 22[7-53] | 11[3-34] | 92[35-203] | 11[4-24] |
| *Disease severity* |  |  |  |  |
| Oxygen saturation, % |  |  |  |  |
| Needed mechanical ventilation | 31(23) | 0(0) | 3(2) | 0(0) |
| Death | 1(1) | 0(0) | 1(1) | 2(2) |
| *Admission site* |  |  |  |  |
| Secondary care center | 50(37) | 148(100) | 74(57) | 99(98) |
| Tertiary care center | 47(34) | 0(0) | 53(40) | 0(0) |
| ICU | 39(29) | 0(0) | 4(3) | 2(2) |
| *Clinical syndrome* |  |  |  |  |
| COPD/Asthma exacerbation | 0(0) | 4(3) | 39(30) | 6(6) |
| LRTI | 80(59) | 70(47) | 87(66) | 85(84) |
| URTI | 56(41) | 74(50) | 5(4) | 10(10) |
| Antibiotic treatment | 64(47) | 87(59) | 117(89) | 97(96) |
| *Study nasal swab PCR* |  |  |  |  |
| 0 microorganism | 16(12) | 55(37) | 67(51) | 58(57) |
| 1 microorganism | 90(66) | 63(43) | 63(48) | 40(40) |
| 2 microorganisms | 21(15) | 24(16) | 1(1) | 3(3) |
| 3 microorganisms | 9(7) | 6(4) | 0(0) | 0(0) |
| *Microorganism* |  |  |  |  |
| Adenovirus | 8(6) | 22(15) | 0(0) | 3(3) |
| Bocavirus | 16(12) | 11(7) | 1(1) | 0(0) |
| Influenza virus | 21(15) | 15(10) | 33(25) | 19(19) |
| Rhinovirus | 34(25) | 28(19) | 12(9) | 10(10) |
| RSV | 65(48) | 33(22) | 9(7) | 9(9) |
| Other* | 14(10) | 20(14) | 10(8) | 5(5) |
| *Expert panel diagnosis* |  |  |  |  |
| Bacterial infection | 32(24) | 43(29) | 84(64) | 59(58) |
| Viral infection | 104(76) | 105(71) | 47(46) | 42(42) |
| Antibiotic overuse | 32(31) | 45(43) | 35(74) | 39(93) |

*Includes coronavirus, human metapneumovirus, and parainfluenza virus.

**Supplemental Fig. 1 Flowchart of microbiologically confirmed patients**

AB - : antibiotics not prescribed, AB +: antibiotics prescribed, RTI: respiratory tract infection, AAU: appropriate antibiotic use, IAU: inappropriate antibiotic use.

**Supplementary Figure 2. Number of antibiotic agents used per country for children and adults with RTIs?.**

NL: The Netherlands, IL; Israel.
